# Effects of Five Different Lactic Acid Bacteria on Bioactive Components and Volatile Compounds of Oat

**DOI:** 10.3390/foods11203230

**Published:** 2022-10-16

**Authors:** Zhishu He, Hao Zhang, Tao Wang, Ren Wang, Xiaohu Luo

**Affiliations:** 1 National Engineering Research Center of Cereal Fermentation and Food Biomanufacturing, Jiangnan University, Wuxi 214122, China; 2 College of Food and Pharmaceutical Sciences, Ningbo University, Ningbo 315211, China

**Keywords:** fermentation, lactic acid bacteria, oats, polyphenols, flavonoids

## Abstract

In this research, oats were fermented with *Lactobacillus plantarum*, *Lactobacillus acidophilus*, *Lactobacillus casei*, *Lactobacillus bulgaricus* and *Streptococcus thermophilus* for 48 h at 37 °C. The purpose of this work was to compare the growth capacities of the five lactic acid bacteria (LAB) in the oat matrix and the effects of fermentation on the contents of the bioactive components of oat, such as *β*-glucan, polyphenols, flavonoids and volatile compounds at different time (0, 4, 8, 12, 24, 36 and 48 h). After 48 h of fermentation, the number of living *L. acidophilus* in oat reached 7.05 × 10^9^ cfu/mL, much higher than that of other strains. *S. thermophilus* retained the greatest *β*-glucan content, and *L. casei* had increased total polyphenol and total flavonoid contents. The proportion of free and bound polyphenols and flavonoids in all samples was changed by microbial action, indicating that forms of polyphenols and flavonoids can be transformed during the fermentation process, and the changes varied with different strains. The samples with *L. plantarum*, *L. acidophilus*, and *L. casei* fermentation contained more alcohols, whereas those with *S. thermophilus* and *L. bulgaricus* fermentation had more aldehydes, which revealed that the composition of volatile components was related to strains. The results indicate that oat substrate is a good medium for LAB growth. This study provides a reference for the use of different strains to achieve different fermentation purposes and a theoretical basis for the further processing of oat and fermented oat beverages.

## 1. Introduction

Oats are grown all over the world, rank seventh in global production behind corn, wheat, rice, barley, sorghum, and millet [1], and are rich in carbohydrates, balanced protein, essential fatty acids, vitamins, and other nutrients [2]. In addition, oats contain many bioactive substances, such as *β*-glucan, polyphenols, and flavonoids. Among them, *β*-glucan is a dietary fiber with hypoglycemic and lipid-lowering effects, which can reduce the risk of obesity, diabetes, and cardiovascular diseases and is of great benefit to human health [3,4]. New research shows that polyphenols can improve intestinal health and plasma inflammation and participate in cell signal transduction pathways owing to their anti-inflammatory, antithrombotic, and antioxidant activities [5]. The oat-based food industry has been promoted by the U.S. Food and Drug Administration’s recommendation to eat more oats because of their high nutritional benefits and the growing emphasis on healthy food [6]. Thus, exploring more oat processing technologies, enriching oat products, and improving economic benefits are the directions of future development.

Ways to improve the nutritional value of grains include cooking, grinding, and fermentation [7]. Bioactive substances in the grain bran tend to bind to the complex structure of the cell wall, which resists traditional crushing processes; however, bioprocessing techniques can effectively solve this problem [8]. Fermentation may be most economical and the simplest way to increase the nutritional value and functional quality of oats [9]. Lactic acid bacteria (LAB) are probiotics commonly used in food production, especially in dairy fermentation. However, exposure to lactose intolerance, milk allergy, and cholesterol content, and the increase in vegetarianism, have led people to pay more attention to the development of fermented products from plant sources [4,10]. In fact, the enzymes produced by LAB, as well as their metabolic capacity, are vital for the synthesis of some beneficial substances and could facilitate their application in the oat beverage market [11]. In the process of fermentation, the glycosidic bonds of some substrates such as polyphenols and flavonoids are hydrolyzed by the *β*-glucosidase of LAB, which may release and increase their concentration, thus improving the potential value of oat the fermented beverage. At the same time, the activation of the peptidase system increases the digestibility of proteins and the level of limiting amino acids. In addition, phytase activity has been increased in low pH environment for better hydrolysis of phytates and enhanced mineral bioaccessibility [12]. There have been some reports of oat-based fermented products, such as a beverage containing 25% of oats fermented with *Lactobacillus plantarum* LP09, which improved polyphenol utilization, antioxidant activity and flavor [13]. Bocchi et al. [14] found that co-fermentation of oat milk by *Lactobacillus* and *Bifidobacterium* decreased phytic acid content and increased the bioavailability of amino acids, polyphenols and vitamins. Martensson et al. [15] combined the prebiotic *β*-glucan of oats with the potential benefits of probiotics to produce a functional product that could lower cholesterol levels.

There is much literature on the development of beverages produced from grains, but studies on the selection of appropriate fermentation conditions and strains are few [13]. In this paper, oats were fermented by different LAB strains, and the growth ability of strains and changes in oat bioactive substances and volatile compounds were monitored to provide guidance for the development of fermented oat beverages.

## 2. Materials and Methods

### 2.1. Materials

Whole oats were bought from a local supermarket (Wuxi, China). *Lactobacillus plantarum* 22158, *Lactobacillus acidophilus* 6089, and *Lactobacillus casei* 6117 were obtained from the China Center of Industrial Culture Collection (Beijing, China). *Lactobacillus delbrueckii* subsp. *bulgaricus* 57004 and *Streptococcus thermophilus* 58013 were obtained from Hubei Provincial Center for the Preservation and Research of Industrial Microbial Strains (Wuhan, China). α-Amylase (3000 U/mL) and glucoamylase (260,000 U/mL) were provided by Jiangsu Boli Biological Products Co., Ltd. (Taizhou, China). de Man–Rogosa–Sharpe (MRS) medium was obtained from Guangdong Huankai Microbial Sci. & Tech. Co., Ltd. (Guangzhou, China). Other analytical chemicals were obtained from China National Medicines Co., Ltd. (Beijing, China).

### 2.2. Preparation of Oat Substrate

Whole oats were cleaned, soaked (1 h), dried, roasted at 170 °C for 15 min, ground by ball mill (SJM-5L, Mitr Instrument Equipment Co., Ltd., Changsha, China) at 4000 rpm for 20 min, added with deionized water according to the solid–liquid ratio of 1:4 (*m*/*v*), liquefied for 30 min (20 U/g α-amylase, 60 °C, pH 6.0), saccharified for 30 min (130 U/g glucoamylase, 60 °C, pH 4.2), homogenized, adjusted to pH 7.0, and pasteurized for reserve use.

### 2.3. Fermentation of Oat Substrates with LAB Strains

*L. plantarum*, *L. acidophilus*, *L. casei*, *L. bulgaricus*, and *S. thermophilus* were activated on the optimal medium (MRS) for two generations. Afterward, 1% LAB (*v*/*v*) was inoculated in 20 mL of oat substrate, then mixed and incubated at 37 °C for 48 h. All samples were shaken gently in the bottle before being tested to achieve a homogeneous sample. The samples fermented with *L. plantarum*, *L. acidophilus*, *L. casei*, *L. bulgaricus*, and *S. thermophilus* were named O-Lp, O-La, O-Lc, O-Lb, and O-St, respectively.

### 2.4. Determination of Viable Counts and pH Value

The bacterial counts of different strains fermented in oat substrate for 0, 4, 8, 12, 24, 36, and 48 h were determined according to the standard plate count method. Sterile saline was used for gradient dilution, and the bacterial solution was inoculated separately onto MRS agar for 48 h and then counted [16].

Additionally, the pH of the samples was measured with a pH meter (PB-10, Sartorius AG, Goettingen, Germany).

### 2.5. Determination of β-Glucan Content

*β*-Glucan content was evaluated by a *β*-glucan assay kit (mixed linkage) (Megazyme, Wicklow, Ireland). A volume of 3 mL of sample was added to 9 mL of 95% ethanol and stood for 5 min. After the supernatant was discarded (1000× *g*, 10 min; Sigma, Lower Saxony, Germany), 10 mL of 50% ethanol was added, dispersed again and the precipitate reserved (1000× *g*, 10 min). Volumes of 0.2 mL of 50% ethanol and 4 mL of sodium phosphate (20 mM, pH 6.5) were added to the precipitate followed by heating in a boiling water bath for 3 min. The tube at was incubated at 50 °C for 5 min and 0.2 mL of lichenase solution (50 U/mL) was added. The reaction was carried out for 1 h with vigorous stirring (3–4 times). After that, 5 mL sodium acetate buffer (200 mM, pH 4.0) was added and the tube allowed to equilibrate to room temperature. The supernatant after centrifugation (0.1 mL; 1000× *g*, 10 min) was hydrolyzed with 0.1 mL *β*-glucosidase solution, and the obtained solution was used for color reaction.

### 2.6. Extraction of Phenolic Compounds

The extraction of phenolic compounds was performed according to the method described by Zhang et al. [17] with some modifications. O-Lp, O-La, O-Lc, O-Lb, and O-St samples (4 mL) with different fermentation time were collected, mixed with 20 mL of 80% ethanol, and placed in an ultrasonic cleaner (KQ-50E, Kun Shan Ultrasonic Instruments Co., Ltd., Kun Shan, China) at 25 °C for 20 min. The obtained liquid was centrifuged at 4000 rpm for 10 min at 4 °C and the above steps repeated three times. The supernatant was collected and concentrated with a vacuum rotary evaporator (RV 10 digital V, IKA, Baden-Wuerttemberg, Germany) at 40 °C. The liquid, containing free phenolics, was kept at a constant volume of 10 mL with methanol and stored under dark condition. The remaining precipitate was added to hexane to remove lipids. Then, the precipitate was hydrolyzed by addition of 20 mL of 4 M NaOH, shaken for 1 h, and adjusted to pH 2.0–3.0 with 6 M HCl. The mixture was extracted with 20 mL of ethyl acetate, followed by ultrasonication for 20 min and centrifugation at 4000 rpm for 10 min. The operation was repeated three times to collect the supernatant, which was vacuum-evaporated at 40 °C. The liquid, containing the bound phenolics, was kept at a constant volume of 10 mL with methanol and stored under dark conditions.

### 2.7. Determination of Phenolic Content

Phenolic content was determined using an adapted and validated method [18] with slight modifications. Briefly, 0.25 mL of extract was mixed with 1 mL of distilled water and 0.25 mL of Folin–Ciocalteu’s phenol reagent was added for reaction for 6 min. Then, 2.5 mL of 7% Na_2_CO_3_ and methanol were added to a total volume of 10 mL. The obtained liquid was incubated for 90 min in darkness at room temperature. Subsequently, absorbance was measured on a spectrophotometer (UV-2100, UNICO, Shanghai, China) at 760 nm. The phenolic compounds were quantified using a gallic acid standard calibration curve. The results were expressed in milligram of gallic acid equivalent (GAE) per 1 L of sample.

### 2.8. Determination of Flavonoid Content

Flavonoid content was measured with reference to the method proposed by Kim et al. [19]. A 1 mL of sample was diluted to 5 mL with 70% ethanol and mixed with 0.3 mL of 5% NaNO_2_ for 5 min. Afterward, 0.3 mL of 10% AlCl_3_·6H_2_O was added to react for 6 min. Finally, 2 mL of 1 M NaOH and 2.4 mL of 70% ethanol were added, and the obtained liquid was incubated for 15 min. The absorbance was read at 420 nm. A rutin standard calibration curve was used for the quantification of flavonoids, and the results were expressed as milligram of rutin equivalent (RE) per 1 L of sample.

### 2.9. Determination of Volatile Components

Samples were incubated at 50 °C for 10 min in a headspace flask (20 mL), extracted continuously with fiber (DVB/CAR/PDMS, 50/30 μm) at 50 °C for 30 min, and desorbed at 250 °C for 5 min.

Two-dimensional gas chromatography–time-of-flight mass spectrometry analysis was conducted using a Pegasus GC-HRT+ 4D high-performance mass spectrometer (LECO Corp., San Jose, CA, USA). Separation was carried out on a MAT-WAX column (30 m × 0.25 mm × 0.25 μm film thickness; Restek, Bellefonte, PA, USA) with a helium (purity > 99.999%) carrier gas, maintaining a constant flow rate of 1 mL/min. The splitless mode was operated. The inlet temperature was set at 250 °C. The oven temperature was initially set at 40 °C for 3 min, then programmed to increase to 230 °C at a rate of 10 °C/min, and held at 230 °C for 6 min. The transmission line temperature and ion source temperature were set to 250 °C. Mass spectra were measured over a range of 33–400 *m*/*z* utilizing an electron energy of 70 eV.

### 2.10. Statistical Analysis

All experiments were conducted in triplicate unless specified. Diagrams were drawn using Origin 2022b (Origin Statistical Software, Northampton, MA, USA). ANOVA with Duncan’s test (*p* < 0.05) was used to analyse the differences between samples in SPSS 24.0 statistics software (SPSS Inc., Chicago, IL, USA).

## 3. Results and Discussion

### 3.1. LAB Growth Curve

**Figure 1:**
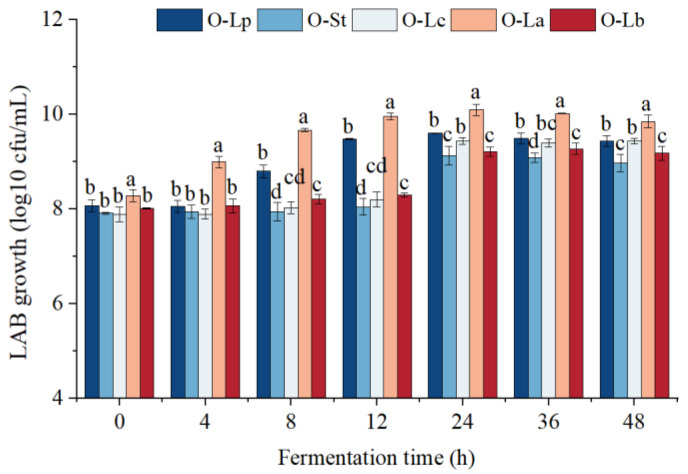
Growth curves of five strains at different incubation time in oat substrate. Different letters indicate significant differences among different strains a given fermentation time (*p* < 0.05). O-Lp, sample fermented with *L. plantarum*; O-La, sample fermented with *L. acidophilus*; O-Lc, sample fermented with *L. casei*; O-Lb, sample fermented with *L. bulgaricus*; O-St, sample fermented with *S. thermophilus.* h, hours; cfu, colony forming units; LAB, Lactic acid bacteria.

The purpose of this work was to monitor and compare the cell viabilities of *L. plantarum*, *L. acidophilus*, *L. casei*, *S. thermophilus*, and *L. bulgaricus* inoculated in oats. As shown in Figure 1, the five strains showed remarkable differences in growth activity during the first 12 h of the fermentation. The differences were caused by the strains’ utilization of carbon and nitrogen sources and adaptability to substrates [20]. *L. acidophilus* and *L. plantarum* had the fastest growth rates. The maximum concentrations of all strains except *L. bulgaricus* were determined after inoculation for 24 h. At this time, *L. acidophilus* entered the stable stage, and the number of viable bacteria reached 1.36 × 10^10^ colony forming units (cfu)/mL. The results showed that *L. acidophilus* and *L. plantarum* had stronger growth abilities in oat substrate than the other strains. The viable counts of the five strains in the lag phase were all higher than the recommended minimum of 10^6^ cfu/mL for probiotic products [21], indicating that all strains could use the nutrients in oats, and the oat substrate was a suitable fermentation substrate. Similar results were shown by Rathore et al. [22]. Who found that *L. plantarum* and *L. acidophilus* grew well in barley and malt substrates. In fact, *L. plantarum*, *L. acidophilus*, and *L. casei* are considered common strains for cereal fermentation [12]. LAB are heterotrophic organisms that lack some biosynthetic processes and have complex nutritional needs [23]. Different LAB can metabolize different carbon sources, and each microorganism shows a specific preference for one or more sugars [24]. This is also why *L. bulgaricus* and *S. thermophilus* have poorer growth abilities in oat compared with other strains, and prefer to use lactose.

### 3.2. Effect of Fermentation on pH

The ability of LAB to produce acid during fermentation is related to whether the growth of miscellaneous bacteria can be inhibited during product storage, and also affects the sensory characteristics of the final product. The results revealed that the five strains could reduce the pH of the substrate to varying degrees during fermentation (Table 1) and finally reach a pH of 3.15–4.25. After 8 h of fermentation, the pH values of *L. plantarum* and *L. acidophilus* decreased rapidly by 27.39% and 27.67%, respectively, which were distinctly different from those of other strains (*p* < 0.05). The maximum decrease in the pH value of the two strains occurred at the same time with the index growth period of the strain. By contrast, *S. thermophilus* has the weakest acid-producing capacity, reaching a pH of 4.25 after 48 h of fermentation, which may be attributed to *L. acidophilus* preferring lactose to glucose as its main energy source [25]. LAB can produce lactic acid through the carbohydrate metabolism pathway. The production of various organic acids may be the main reason for the decrease in pH [26]. The above results are supported by the study of Mirmohammadi et al. [27]. They concluded that the pH values of different substrates decrease rapidly within 12 h after fermentation by LAB. The strains used in the production of fermented cereal beverages and fermentation time affect the pH of the product.

### 3.3. Effect of Fermentation on β-Glucan Content

**Figure 2:**
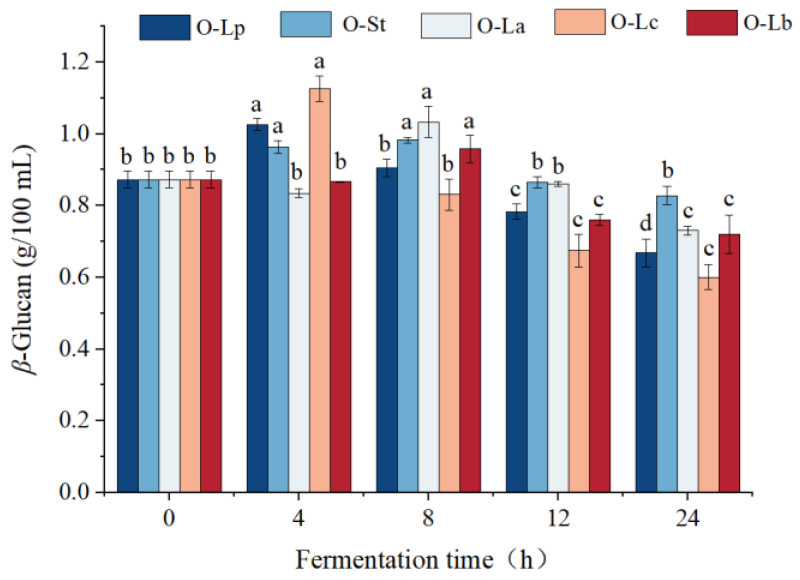
Changes of *β*-glucan content in oats fermented by different strains at different times. Different letters indicate significant differences among different fermentation time a given fermentation strain (*p* < 0.05). O-Lp, sample fermented with *L. plantarum*; O-La, sample fermented with *L. acidophilus*; O-Lc, sample fermented with *L. casei*; O-Lb, sample fermented with *L. bulgaricus*; O-St, sample fermented with *S. thermophilus*. h, hours.

Our work aimed to study the effect of LAB on *β*-glucan content during oat fermentation. The *β*-glucan contents of the five strains shown in Figure 2 increased at the initial fermentation stage, which may be related to the fact that insoluble *β*-glucan was degraded to soluble *β*-glucanase by LAB [28], and then LAB consumed a large amount of carbohydrates for proliferation. *β*-Glucan can also provide growth substrates (prebiotics) for some LAB. Therefore, the contents of all samples were significantly decreased at 8–12 h of fermentation (*p* < 0.05), but the decreases were different. The sample fermented with *S. thermophilus* for 24 h decreased the *β*-glucan content by 5.09% compared with the unfermented sample; thus, O-St had the most *β*-glucan content. Evidence shows that *S. thermophilus* TKM3 KKP2030p does not grow well in oat–banana matrix and does not utilize *β*-glucan [29]. This finding was supported by the results of the viable count experiment and *β*-glucan content in the present study. Studies showed that the change in *β*-glucan content during fermentation is related to the strain type. Interestingly, Sims et al. [30] concluded that *β*-glucan oligosaccharide supported *L. rhamnosus* growth, but *B. lactis* and *L. acidophilus* did not grow on this substrate. Therefore, the different utilization of *β*-glucan by different strains in the oat matrix may be the reason for the inconsistent decrease in *β*-glucan content. These data provide guidance for the development of fermented oat beverages. Strains that do not ferment *β*-glucan can be selected to maximize the potential of probiotics. 

### 3.4. Effect of Fermentation on Phenolic Content

**Figure 3:**
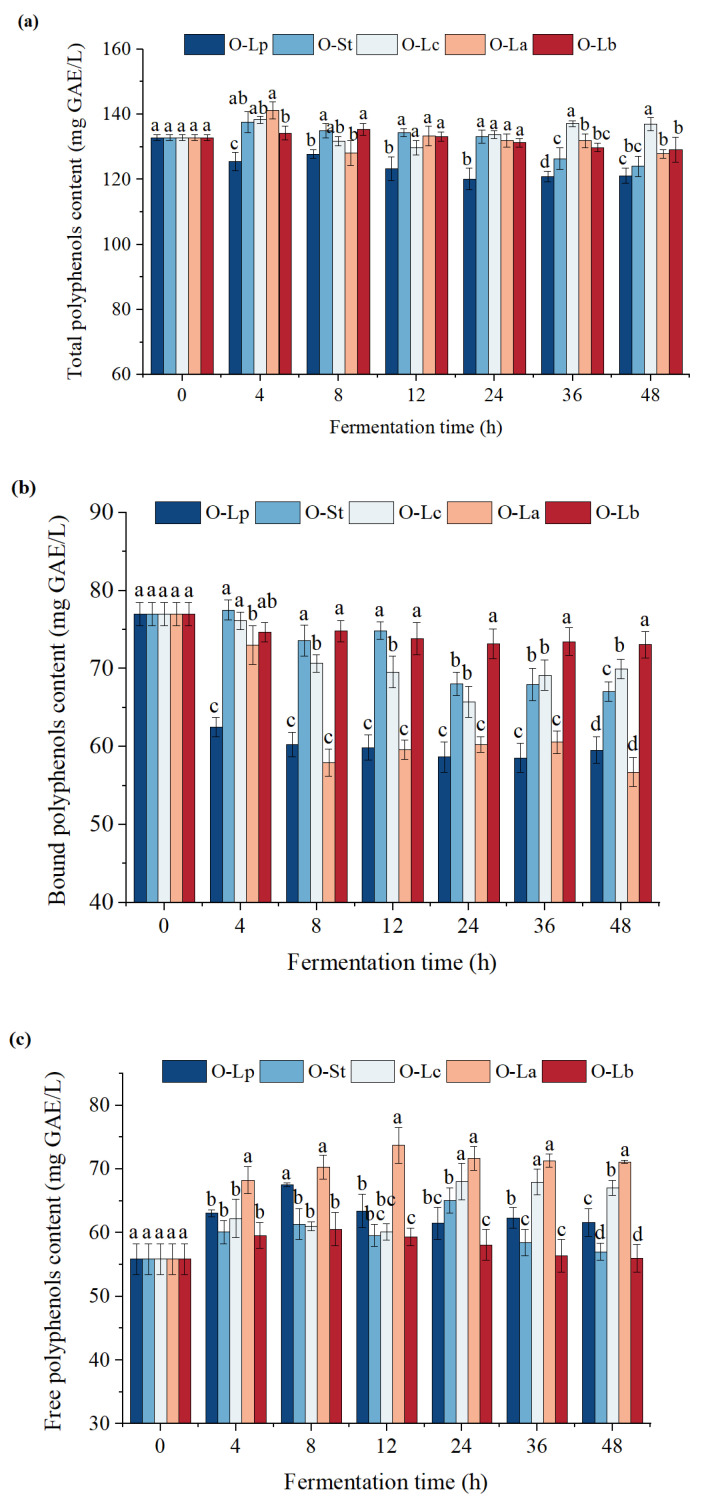
Contents of total polyphenols (**a**), bound polyphenols (**b**) and free polyphenols (**c**) in samples of different strains at different fermentation times. Different letters indicate significant differences among different strains a given fermentation time (*p* < 0.05). O-Lp, sample fermented with *L. plantarum*; O-La, sample fermented with *L. acidophilus*; O-Lc, sample fermented with *L. casei*; O-Lb, sample fermented with *L. bulgaricus*; O-St, sample fermented with *S. thermophilus*. h, hours.

In fermented products, the glycoside form of polyphenols can be transformed into the aglycone form by microorganisms to improve their bioavailability in the intestine and perform their beneficial functions better. Changes in total phenolic content (TPC), bound phenolic content (BPC), and free phenolic content (FPC) during oat fermentation by LAB are depicted in Figure 3a–c, respectively. The results indicate that a strain has strict specificity in phenolic acid metabolism/degradation/hydrolysis as reported in the literature [31]. For example, as shown in the figure, the TPCs of some strains were distinctly different at the given fermentation time (*p* < 0.05). Compared with the unfermented samples, the TPCs of all fermented samples showed a decreasing trend at the later stage of fermentation, particularly after 12 h, except for O-Lc. However, after 48 h of fermentation, only the TPC of O-Lc increased by 3.14%. Similar results were found by Li et al. [32], who fermented jujube juice with *L. plantarum* and *L. casei* to increase TPC. Other results showed that fermentation could also reduce phenolic content. Moreover, extractability was reduced by the self-polymerization of phenolic compounds and/or the interaction with other macromolecules (such as amino acids and starch) [33]. In addition, compounds are also be transformed and degraded into other healthy monomers [8]. 

Figure 3b,c show that the FPCs of the five strains were higher than that of the control sample within 24 h of fermentation, whereas the BPCs had the opposite trend. Additionally, an increase in the FPC of each sample was often accompanied by a decrease in BPC. This phenomenon can be explained by the transformation of BPC into FPC due to the metabolic activities of microorganisms. LAB grow rapidly in the early stage of fermentation and can use sugar and protein to partially release BPC. In addition, esterase, decarboxylase, and *β*-glucanase are produced in the proliferation process. Among them, *β*-glucanase hydrolyzes the *β*-glycosidic bond of conjugated phenolic compounds [34], resulting in the release of conjugated phenolic compounds and an increase in FPC [35]. Ferulic acid esterase also releases BPC from the grain cellulose matrix into a free form. At different fermentation times, the FPC of O-Lc was always higher than those of other samples (*p* < 0.05), especially at 12 h, when it could reach 73.70 mg GAE/L, which was 1.32 times higher than that of the control samples. The BPC in O-Lc was remarkably lower than those of O-St, O-Lc, and O-Lb. In addition to glucanase activity, the ability of bacterial strains to degrade phenol esters or tannins, different induced phenol decarboxylase activities, and different substrate acidity may also contribute to this result [36]. Overall, fermentation can change the phenolic contents in the samples. However, whether the compositions of free and bound monomeric phenolic compounds change needs further verification. 

### 3.5. Effect of Fermentation on Flavonoid Content

**Figure 4:**
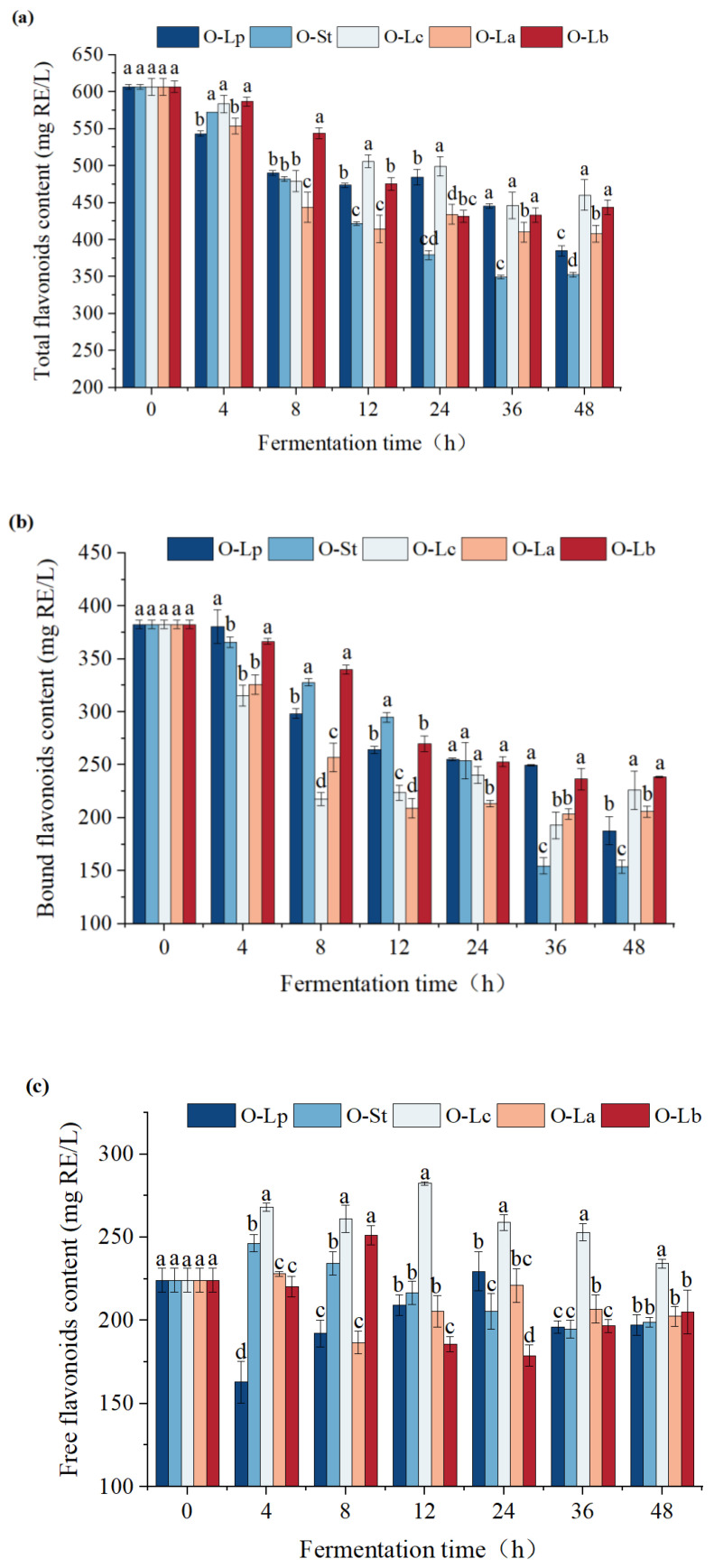
Contents of total flavonoids (**a**), bound flavonoids (**b**) and free flavonoids (**c**) in samples of different strains at different fermentation time. Different letters indicate significant differences among different strains a given fermentation time (*p* < 0.05). O-Lp, sample fermented with *L. plantarum*; O-La, sample fermented with *L. acidophilus*; O-Lc, sample fermented with *L. casei*; O-Lb, sample fermented with *L. bulgaricus*; O-St, sample fermented with *S. thermophilus*. H, hours.

Flavonoids are important natural organic compounds that exist widely in nature. Most flavonoids have strong biological activities, which has aroused research interest. Flavonoids occupy an increasingly important position in daily diet and disease treatment owing to their extensive pharmacological effects and low toxicity. The content change of flavonoids during oat fermentation is shown in Figure 4. The content of total flavonoids in oats fermented by different strains showed a downward trend within 48 h as shown in Figure 4a. This result may be related to the large reduction of conjugated flavonoids. According to previous reports, flavonoids can be transformed into free forms in the fermentation process of soybean and tea, but a decrease in the total amount may not indicate a decrease in biological activity. Bound and free flavonoids decreased during the fermentation of corn and other grains [37]. Researchers attributed this to the metabolism of these compounds during microbial fermentation, such as the degradation/polymerization of flavonoids into dihydroxy flavonoids analogues and the activity changes of enzymes, including glycosidase, glycosyltransferase, tannase, esterase, and hydrolase [34]. In addition, some reactions may induce flavonoids to transform into other metabolites through flavonoid methylation, glycosylation, flavonoid deglycosylation, and flavonoid–sulfuric acid conjugation.

Figure 4b,c shows that after 4 h of fermentation, the free flavonoids in the samples of the other four bacteria except O-Lp were increased, and the bound flavonoids were decreased. O-Lp had the opposite trends initially but gradually had the same trends in the later fermentation time. This outcome could be related to the depolymerization of bound flavonoids and the formation of soluble free flavonoids. Interestingly, we found that at each fermentation time point, the free flavonoid content of O-Lc was distinctly higher than in those of other strains (*p* < 0.05), reaching 202.60 mg RE/L at 48 h, an increase of 20.47% compared with the unfermented sample. Flavonoid glycosides may be consumed during fermentation and released in the form of aglycones. LAB can convert flavonoid glycosides in Cudrania tricuspidate leaves into flavonols, quercetin, and kaempferol [38].

Current studies on the biotransformation of flavonoids in food fermentation processes are few. Xu et al. [39] fermented milk containing *Scutellaria baicalensis* with *Lactobacillus brevis* and found that baicalin and wogonoside were converted into their aglycone forms, baicalein and wogonin, respectively, which have higher biological activities. The biotransformation mechanism of flavonoids in LAB-fermented food remains to be further studied. 

### 3.6. Effect of Fermentation on Volatile Components

The volatile components of oat fermented by different LAB strains for 48 h are shown in Table 2. The volatile components included 10 alcohols, 10 aldehydes, 7 acids, 15 ketones, 7 esters, 11 furan derivatives, 8 hydrocarbons, and 1 terpene. Oat is prone to oxidative rancidity and deterioration during processing, storage, and circulation, which is related to the high fat content of oat, especially the high percentage of unsaturated fatty acids and the large amount of lipase with high activity in the endosperm. The oxidative cleavage of oleic or linoleic acid during the contact between unsaturated fatty acids and lipase in oat crushing or milling produces hexanal and nonanal. Nonanal production may be caused by the loss of a hydrogen from the 10th carbon of the oleic acid chain, followed by the absorption of a hydrogen peroxyfree group (OOH) and subsequent fracture. The cleavage of the 13-hydroperoxide after the oxygenation of linoleic acid chain may lead to the formation of hexaldehyde. The fatty oxygenase-specific oxidation of 9-hydroperoxides can form 2-pentylfurans, which have greeny, beany, and buttery aromas, whereas 1-octen-3-ol may arise from the 10-hydroperoxide of linoleic acid. This finding was confirmed by the fact that 1-octene-3-ol, hexanal, and nonanal had high concentrations in the unfermented samples as presented in Table 2, revealing that partial oxidation occurred before fermentation. At the same time, we observed that 2-pentylfuran, as one of the important volatile compounds, was detected in each fermented sample, and was significantly higher than that in the fermented sample (*p* < 0.05). This indicates that oxidation also occurred during fermentation.

No pentanal, hexanal and heptanal were detected in O-Lc and O-La. This result was very similar to the conclusion of Lee et al. [40], who fermented oats with *L. paracasei* and found that hexanal content decreased considerably within the first 2 h and was completely undetectable at 24 h. This outcome was due to the fact that microbial action can convert aldehydes into alcohols and acids. In our experiment we observed the fact that 1-pentanol, 1-hexanol, 1-heptanol, acetic acid and hexanoic acid increased correspondingly. Overall, after LAB fermentation, the contents of aldehydes in the sample decreased, and the contents of alcohols, acids, and ketones increased. However, there were distinct differences in flavor components among fermented samples with different strains. As can be seen from Table 2, the contents of alcohols and acids in O-Lp, O-Lc and O-La were higher than those in O-St and O-Lb by up to 5.86 and 2.55 times (O-La compared to O-Lb). In contrast, the contents of aldehydes and esters in O-St and O-Lb were higher than those of O-Lp, O-Lc and O-La, up to 2.85 and 9.61 times (O- St compared to O-La).This difference may be closely related to carbohydrate and amino acid metabolism. The taste characteristics of amino acids and their catabolic by-products during LAB fermentation make an important contribution to the flavor of cereal-based foods [41]. O-St and O-Lb contained lesser 1-hexanol, benzyl alcohol (fruity and slightly floral), phenylethanol (floral and slightly rose), acetic acid, hexanoic acid (sweaty and cheesy), valeric acid, and nonanoic acid and more abundant hexanal (grassy, tallow, fatty), heptanal octanal (fatty, greeny), nonanal (fatty, citrus, greeny), and esters (floral and fruit flavor). Alcohols were predominant in O-Lp, O-Lc, and O-La. 2,3-Butanedione and 3-hydroxy-2-butanone were only present in O-Lc and O-La, which showed a buttery odor. Limonene, which has a citrus-like aroma, was detected in all samples but at low concentrations.

Fermented oats contain volatile components, such as 1-hexanol, hexanal, nonanal, acetic acid and 2-pentylfuran. These components are also considered the key flavor compounds in Lactobacillus-fermented foods and influence the organoleptic properties of fermented products.

## 4. Conclusions

In summary, oat substrate is a suitable fermentation substrate. *L. plantarum*, *L. casei*, *L. acidophilus*, *L. bulgaricus*, and *S. thermophilus* could grow well in oat substrate, and the number of viable bacteria could reach 10^6^ cfu/mL even at the late fermentation stage. *L. acidophilus* showed the strongest growth ability in oat substrate, and the number of live bacteria was the highest. Soaking oats for 1 h prior to fermentation made them more conducive to subsequent cleaning and absorption of water without significantly affecting the bioactive components. *L. plantarum* and *L. acidophilus* had the strongest acid-producing capacity during the fermentation process, and *S. thermophilus* retained the most *β*-glucan content after 48 h of fermentation. Moreover, the contents of total polyphenols and total flavonoids in oats varied with different strains, among which O-Lc was the highest. Aldehydes were predominant in O-Lb and O-St, but alcohols were predominant in O-Lp, O-Lc, and O-La. In conclusion, different strains used in oat fermentation have different effects. Therefore, suitable LAB can be selected in future research to improve beneficial ingredients through microbial-mediated biotransformation, providing guidance for the research and development of cereal fermentation products and other options for vegetarians and lactose-intolerant people.

## Figures and Tables

**Table 1:** Changes of pH of oat substrate during 48 h fermentation by different lactic acid bacteria.

Time (h)	O-Lp	O-La	O-St	O-Lb	O-Lc
0	7.01 ± 0.01 a	7.01 ± 0.03 a	7.01 ± 0.03 a	7.01 ± 0.03 a	7.01 ± 0.03 a
4	6.23 ± 0.02 d	6.24 ± 0.01 d	6.76 ± 0.01 a	6.54 ± 0.01 b	6.35 ± 0.04 c
8	5.09 ± 0.08 d	5.07 ± 0.03 d	6.27 ± 0.02 a	5.95 ± 0.04 b	5.85 ± 0.02 c
12	4.27 ± 0.05 d	4.12 ± 0.03 e	5.85 ± 0.06 a	5.46 ± 0.07 b	5.36 ± 0.05 c
24	3.54 ± 0.05 c	3.5 ± 0.06 c	5.17 ± 0.06 a	4.48 ± 0.01 b	4.56 ± 0 b
36	3.3 ± 0.06 d	3.23 ± 0.01 e	4.48 ± 0.02 a	3.79 ± 0.01 b	3.55 ± 0.02 c
48	3.15 ± 0.01 d	3.14 ± 0.03 d	4.25 ± 0.01 a	3.76 ± 0.04 b	3.22 ± 0.03 c

Different lowercase letters on the same line indicate significant differences among samples (*p* < 0.05). O-Lp, sample fermented with *L. plantarum*; O-La, sample fermented with *L. acidophilus*; O-Lc, sample fermented with *L. casei*; O-Lb, sample fermented with *L. bulgaricus*; O-St, sample fermented with *S. thermophilus*. h, hours.

**Table 2:** Changes of volatile compounds in oat samples fermented by different lactic acid bacteria for 48 h.

Volatile Compounds	RT (s)	Control (%)	O-Lp (%)	O-Lc (%)	O-La (%)	O-St (%)	O-Lb (%)
Alcohols							
2-Butanol	411.88	ND	ND	ND	ND	0.33 ± 0.02 a	ND
1-Pentanol	603.74	ND	0.49 ± 0.03 b	0.46 ± 0.04 b	0.59 ± 0.02 a	0.29 ± 0.02 d	0.34 ± 0.02 c
1-Hexanol	696.64	0.09 ± 0.01 d	4.81 ± 0.32 a	1.96 ± 0.23 b	5.22 ± 0.41 a	0.63 ± 0.06 c	0.10 ± 0.03 d
1-Octen-3-ol	778.63	0.46 ± 0.03 b	0.39 ± 0.02 c	0.44 ± 0.03 bc	0.75 ± 0.03 a	0.38 ± 0.05 c	0.43 ± 0.03 bc
1-Heptanol	782.84	0.25 ± 0.02 c	0.69 ± 0.03 a	0.35 ± 0.05 b	0.76 ± 0.08 a	0.18 ± 0.03 c	0.19 ± 0.04 c
1-Octanol	863.64	0.46 ± 0.03 d	0.95 ± 0.04 b	0.58 ± 0.02 c	1.08 ± 0.02 a	0.40 ± 0.04 e	0.47 ± 0.03 d
2-Octen-1-ol, (Z)-	908.51	0.05 ± 0.01 b	0.04 ± 0.00 b	0.03 ± 0.01 b	0.11 ± 0.01 a	0.03 ± 0.00 b	0.03 ± 0.02 b
1-Nonanol	939.49	ND	0.99 ± 0.12 a	0.32 ± 0.02 b	0.96 ± 0.17 a	ND	0.04 ± 0.02 c
Benzenemethanol	1098.00	0.02 ± 0.00 b	0.12 ± 0.05 a	0.09 ± 0.03 a	0.12 ± 0.02 a	0.02 ± 0.01 b	0.03 ± 0.01 b
Benzeneethanol	1121.35	0.02 ± 0.00 b	0.09 ± 0.01 a	0.08 ± 0.01 a	0.09 ± 0.02 a	0.02 ± 0.00 b	0.02 ± 0.00 b
Aldehydes							
Pentanal	321.59	0.35 ± 0.09 a	ND	ND	ND	0.25 ± 0.05 a	0.28 ± 0.02 a
Hexanal	431.62	4.63 ± 0.34 a	0.06 ± 0.01 c	ND	ND	3.34 ± 0.37 b	3.84 ± 0.30 b
Heptanal	542.25	0.60 ± 0.04 b	ND	ND	ND	0.42 ± 0.02 c	0.74 ± 0.02 a
2-Hexenal, (E)-	578.54	0.10 ± 0.02 a	0.04 ± 0.00 b	ND	0.04 ± 0.01 b	0.09 ± 0.02 a	0.11 ± 0.01 a
Octanal	644.40	1.81 ± 0.25 a	ND	ND	0.36 ± 0.04 c	0.95 ± 0.03 b	0.88 ± 0.05 b
2-Heptenal, (E)-	679.51	0.41 ± 0.02 a	0.27 ± 0.04 c	0.14 ± 0.01 d	0.35 ± 0.02 b	0.26 ± 0.05 c	0.30 ± 0.04 bc
Nonanal	738.364	2.32 ± 0.32 a	0.95 ± 0.04 c	0.95 ± 0.03 c	1.10 ± 0.05 c	1.96 ± 0.12 b	2.48 ± 0.31 a
2 Octenal	771.40	0.19 ± 0.02 a	0.14 ± 0.01 c	0.06 ± 0.02 d	0.16 ± 0.02 bc	0.02 ± 0.00 e	0.17 ± 0.01 ab
Benzaldehyde	852.46	ND	ND	ND	0.51 ± 0.03 b	0.56 ± 0.04 a	ND
2-Nonenal, (E)-	856.75	0.55 ± 0.03 a	0.44 ± 0.13 b	0.05 ± 0.04 c	0.40 ± 0.01 b	0.47 ± 0.03 ab	0.56 ± 0.01 a
Acids							
Acetic acid	788.20	ND	2.31 ± 0.34 a	1.62 ± 0.12 b	2.54 ± 0.24 a	0.39 ± 0.09 c	0.56 ± 0.11 c
Hexanoic acid	1073.15	0.41 ± 0.02 d	0.73 ± 0.05 c	1.37 ± 0.12 a	0.88 ± 0.03 b	0.50 ± 0.01 d	0.70 ± 0.02 c
Pentanoic acid	1073.79	ND	0.25 ± 0.01 b	0.08 ± 0.01 c	0.88 ± 0.07 a	ND	0.05 ± 0.02 c
Heptanoic acid	1141.51	0.31 ± 0.02 a	0.28 ± 0.03 ab	0.24 ± 0.01 bc	0.23 ± 0.03 c	0.21 ± 0.02 c	0.23 ± 0.02 c
Octanoic acid	1206.61	0.13 ± 0.02 e	0.65 ± 0.03 a	0.44 ± 0.03 c	0.53 ± 0.02 b	0.28 ± 0.02 d	0.41 ± 0.01 c
Nonanoic acid	1268.57	ND	0.12 ± 0.01 a	0.11 ± 0.01 a	0.11 ± 0.00 a	0.06 ± 0.00 b	ND
Decanoic acid	1327.65	ND	0.01 ± 0.00 b	ND	ND	ND	0.07 ± 0.01 a
Ketones							
2,4-Pentanedione	148.80	ND	ND	ND	ND	0.24 ± 0.04 a	ND
2-Propanone	188.36	ND	0.07 ± 0.00 d	0.09 ± 0.01 c	0.09 ± 0.01 c	0.17 ± 0.00 b	0.19 ± 0.01 a
2- butanone	245.99	ND	ND	0.03 ± 0.00 a	ND	ND	ND
2-Pentadecanone	260.50	ND	ND	0.05 ± 0.00 a	ND	ND	ND
2,3-Butanedione	322.60	ND	ND	0.39 ± 0.02 a	0.11 ± 0.01 b	ND	ND
2,3-Pentanedione	409.87	ND	ND	ND	ND	0.10 ± 0.01 a	ND
2-Heptanone	539.31	0.58 ± 0.04 b	0.54 ± 0.07 b	0.61 ± 0.03 ab	0.69 ± 0.07 a	0.54 ± 0.01 b	0.59 ± 0.03 b
3-Octanone	610.63	ND	0.04 ± 0.01 ab	0.03 ± 0.00 b	0.05 ± 0.01 a	0.03 ± 0.00 b	ND
2-Octanone	640.36	ND	ND	0.15 ± 0.01 a	0.16 ± 0.02 a	ND	0.14 ± 0.01 a
Methoxy-1-phenyl-2-propanone	641.12	ND	ND	0.71 ± 0.02 a	0.16 ± 0.03 b	ND	ND
2-Butanone, 3-hydroxy-	644.82	ND	ND	0.56 ± 0.03 a	0.36 ± 0.02 b	ND	ND
1-Octen-3-one	656.24	0.09 ± 0.00 c	0.13 ± 0.01 b	0.07 ± 0.00 d	0.19 ± 0.01 a	0.06 ± 0.00 d	ND
2-Propanone, 1-hydroxy-	659.94	0.01 ± 0.00 b	0.02 ± 0.01 ab	0.02 ± 0.00 ab	0.03 ± 0.01 a	ND	0.01 ± 0.00 b
3,5-octadiene-2-one	843.73	0.09 ± 0.02 a	0.10 ± 0.03 a	0.09 ± 0.02 a	0.10 ± 0.01 a	0.01 ± 0.01 b	ND
6-Undecanone	845.96	0.02 ± 0.01 a	ND	0.02 ± 0.00 a	ND	ND	ND
Esters							
Formic acid, pentyl ester	473.96	0.07 ± 0.02 a	ND	0.05 ± 0.01 a	ND	ND	0.06 ± 0.01 a
Heptanoic acid, methyl ester	639.94	1.08 ± 0.04 a	0.32 ± 0.09 b	ND	ND	0.32 ± 0.05 b	ND
1-Cyclopropylpentyl acetate	688.58	ND	0.23 ± 0.03 a	0.19 ± 0.03 a	0.19 ± 0.02 a	0.19 ± 0.01 a	0.22 ± 0.05 a
2-etynyl-3-ethyl-2-buten-4-olide	738.56	ND	ND	ND	ND	1.97 ± 0.32 b	2.48 ± 0.51 a
Hexanoic acid, pentyl ester	831.13	ND	0.02 ± 0.00 a	0.01 ± 0.00 a	0.02 ± 0.01 a	0.01 ± 0.00 a	0.02 ± 0.01 a
2-methylpropyl heptanoate	845.64	ND	0.03 ± 0.01 a	ND	0.03 ± 0.00 a	0.02 ± 0.00 a	0.02 ± 0.01 a
Octadecanoic acid, methyl ester	1296.47	0.03 ± 0.01 ab	0.05 ± 0.02 a	0.04 ± 0.00 ab	0.03 ± 0.00 ab	0.02 ± 0.00 b	0.04 ± 0.01 ab
Furan derivatives							
Furan, 2,3-dihydro-	124.44	ND	0.88 ± 0.06 a	0.81 ± 0.09 a	ND	ND	ND
Furan, 2-methyl-	222.88	ND	ND	ND	0.08 ± 0.02 a	ND	ND
Furan, 2-ethyl-	295.38	ND	0.08 ± 0.01 a	0.02 ± 0.00 b	0.09 ± 0.01 a	0.07 ± 0.00 a	0.08 ± 0.02 a
2-Propylfuran	377.78	ND	0.07 ± 0.02 a	ND	0.08 ± 0.01 a	0.06 ± 0.01 a	0.07 ± 0.00 a
2-Butylfuran	483.20	0.10 ± 0.00 a	ND	0.07 ± 0.01 b	0.11 ± 0.01 a	0.07 ± 0.00 b	0.11 ± 0.02 a
2-tert-Butoxytetra-hydrofuran	515.08	0.11 ± 0.02 b	ND	0.19 ± 0.05 a	ND	ND	ND
Furan, 2-pentyl-	585.34	ND	2.20 ± 0.09 b	2.74 ± 0.13 a	2.27 ± 0.31 b	2.08 ± 0.08 b	2.66 ± 0.22 a
Furan, 2-(1-pentenyl)-, (E)-	739.45	ND	ND	0.04 ± 0.01 b	0.08 ± 0.02 b	ND	2.48 ± 0.27 a
2-Heptyl furan	769.09	0.01 ± 0.01 a	0.01 ± 0.00 a	0.000 ± 0.00 a	0.01 ± 0.01 a	0.01 ± 0.00 a	0.01 ± 0.00 a
Furan, 2-(methoxymethyl)	981.74	0.14 ± 0.04 a	0.11 ± 0.03 ab	0.05 ± 0.01 c	0.08 ± 0.01 bc	0.13 ± 0.02 ab	0.14 ± 0.03 a
2-Phenylfuran	1087.83	0.06 ± 0.02 a	0.07 ± 0.01 a	0.05 ± 0.02 a	0.06 ± 0.01 a	0.06 ± 0.01 a	0.06 ± 0.02 a
Hydrocarbons							
Pentane	127.57	ND	0.48 ± 0.03 a	0.45 ± 0.03 a	0.43 ± 0.02 a	0.49 ± 0.05 a	0.31 ± 0.04 b
Heptane	176.43	0.07 ± 0.02 b	0.41 ± 0.12 a	ND	ND	0.06 ± 0.02 b	0.09 ± 0.01 b
Undecane	338.38	0.01 ± 0.00 b	0.01 ± 0.00 b	0.01 ± 0.00 b	0.09 ± 0.02 a	0.01 ± 0.00 b	0.01 ± 0.00 b
Dodecane	543.61	ND	0.25 ± 0.04 a	ND	0.16 ± 0.03 b	ND	ND
Pentadecane	546.45	0.17 ± 0.02 ab	ND	0.14 ± 0.01 b	ND	0.19 ± 0.03 a	0.16 ± 0.02 ab
Tetradecane	643.72	ND	0.36 ± 0.03 a	0.01 ± 0.01 b	0.36 ± 0.02 a	0.04 ± 0.00 b	ND
Nonadecane	893.62	0.05 ± 0.01 a	0.02 ± 0.01 c	0.05 ± 0.00 a	0.05 ± 0.01 a	0.03 ± 0.00 bc	0.04 ± 0.01 ab
Terpenes							
Limonene	548.57	0.01 ± 0.00 c	0.03 ± 0.01 b	0.05 ± 0.02 ab	0.03 ± 0.01 b	0.06 ± 0.01 a	0.05 ± 0.00 ab

Different lowercase letters indicate significant differences between different lactic acid bacteria fermented samples (*p* < 0.05). O-Lp, sample fermented with *L. plantarum*; O-La, sample fermented with *L. acidophilus*; O-Lc, sample fermented with *L. casei*; O-Lb, sample fermented with *L. bulgaricus*; O-St, sample fermented with *S. thermophilus*. ND, not detected; RT: retention time; h, hours.

## Data Availability

The data presented in this study are available on request from the corresponding author.
